# Evaluating virtual reality technology in psychotherapy: impacts on anxiety, depression, and ADHD

**DOI:** 10.3389/fpsyt.2024.1480788

**Published:** 2024-12-18

**Authors:** Peng Wang, Xiaowen Ai, Xiyang Zhang, Fei Ma, Yan Zhuang, Suogang Wang

**Affiliations:** ^1^ Neuroengineering Laboratory, School of Biomedical Engineering and Technology, Tianjin Medical University, Tianjin, China; ^2^ School of Big Data Intelligent Diagnosis & Treatment Industry, Taiyuan University, Taiyuan, China; ^3^ Medical Psychology Department, Taiyuan Mental Hospital, Taiyuan, China

**Keywords:** VR-CBT, anxiety, depression, ADHD, artificial intelligence

## Abstract

**Background:**

Mental health issues pose a significant challenge for medical providers and the general public. The World Health Organization predicts that by 2030, mental health problems will become the leading cause of global disease burden, highlighting the urgent need for effective mental health interventions. Virtual reality-cognitive behavioral therapy (VR-CBT) has emerged as a promising treatment for neuropsychiatric disorders, offering immersive and engaging therapeutic experiences.

**Objective:**

This study aims to assess the advancements in applying virtual reality (VR) technology for diagnosing and treating mental illnesses such as anxiety, depression, and attention deficit hyperactivity disorder (ADHD). It also explores the integration of artificial intelligence (AI) with VR technology in mental health treatment and introduces the CBT-CHAT Treatment Framework to enhance cognitive behavioral therapy.

**Method:**

A systematic search was conducted across the databases of Google Scholar, PubMed, and Web of Science, followed by a scoping review. Duplicates were removed using the EndNote literature management software. Each article was then carefully read and evaluated for its research content, methodology, results, and contribution to the application of VR technology in mental health domains.

**Results:**

The search retrieved 686 articles, and after applying inclusion and exclusion criteria, 32 articles were ultimately selected. These articles covered the application of VR in anxiety, depression, and ADHD. The research results indicate that VR shows promising outcomes in the diagnosis, treatment, and rehabilitation of anxiety, depression, and ADHD, particularly in the diagnosis, treatment, and rehabilitation of ADHD.

**Conclusion:**

The previously published studies consistently demonstrate that VR is an effective tool for supporting the treatment of mental illnesses across various settings and recommend its incorporation into clinical practice.

## 1 Introduction

### 1.1 Background

Mental health conditions pose significant challenges to society, healthcare providers, and health systems. The academic and employment pressures faced by graduates, the work and life pressures of the general population, and unpredictable pandemics exacerbate these issues (1). Mental health services are striving to meet the needs of users but are unable to cater to the large number of individuals requiring care. The World Health Organization (WHO) predicts that by 2030, mental disorders will become the leading cause of the global burden of disease (2). Furthermore, the WHO estimates that anxiety disorders lead to an economic loss of approximately 1 trillion US dollars in productivity costs annually (3). Therefore, a safe and effective non-pharmacological approach to mental illness intervention is crucial. While some reviews have explored the application of VR in treating anxiety, depression, and attention deficit hyperactivity disorder (ADHD) (4, 5), they have neither primarily focused on depression and ADHD as the main assessment targets, nor have they thoroughly examined the integration of artificial intelligence (AI) in cognitive behavioral therapy (CBT). Additionally, we conducted an exploratory analysis that employed VR scenarios and CBT techniques to improve ADHD. Furthermore, we proposed the CBT-CHAT model framework, aimed at enhancing the efficacy of CBT.

### 1.2 Cognitive behavioral therapy

CBT is a form of psychotherapy that helps individuals identify and change destructive or disturbing thought patterns that negatively impact behavior and emotions (6–8). It is the most empirically supported treatment method and has been shown to effectively assist patients in overcoming a wide range of mental health issues, including anxiety and depression.

### 1.3 VR and VR-CBT

VR is a rapidly developing technology with promising applications in various therapeutic settings. VR creates immersive and interactive computer-generated environments, where users wear headsets to display these virtual worlds and interact with them using specialized controllers or body movements. This immersive nature allows therapists to create safe and controlled environments to practice coping skills, address anxieties, and challenge negative thought patterns (9). VR exposure therapy (VRET) is a modern type of exposure therapy that follows the same procedures as traditional exposure therapy, but the feared objects or situations are presented within a virtual environment. The virtual environment provides therapists with greater control to customize, replicate, and adjust multiple treatment parameters according to the patient’s needs, offering a level of customization that cannot be achieved in traditional therapy. In recent years, the rapid advancement of VR technology has highlighted the distinct advantages of VR-CBT (8). The immersive and personalized nature of VR offers patients a richer therapeutic experience. By establishing a highly controlled, secure, and immersive virtual environment, VR-CBT paves the way for tailored therapeutic interventions.

### 1.4 Objectives

This study follows the scoping review methodology proposed by Arksey and O’Malley (10). In this scoping review, we aim to address three main questions: (1) Which VR technologies and interventions have recently been explored in the research on anxiety, depression and ADHD? (2) Is VR-CBT effective in the intervention for patients with anxiety, depression, and ADHD? (3) What recent research exists on the combination of VR and AI in the intervention for anxiety, depression and ADHD?

As technology rapidly advances, it is crucial to understand the current state-of-the-art technologies being used, especially in interdisciplinary fields such as VR in mental health. Therefore, the first question focuses on the application of VR technology in anxiety, depression, and ADHD. The second question systematically examines the effectiveness of VR-CBT in combination with the treatment of mental disorders. Lastly, the third question explores the emerging trends and applications of AI combined with VR-CBT in mental disorder interventions and proposes a CBT-CHAT treatment framework based on a large language model (LLM) to enhance CBT.

## 2 Methods

### 2.1 Search strategy and data sources

In our methodology, we designed a focused search strategy to identify the most pertinent literature investigating the application of Virtual Reality (VR) in supporting and enhancing mental health outcomes, with a particular emphasis on anxiety, depression, and ADHD. Utilizing Google Scholar, PubMed, and Web of Science (WOS) as our primary databases, we capitalized on their broad reach across disciplines and journals. Our search was refined by strategically selecting keywords such as “Anxiety”, “Depression”, “ADHD”, and “Virtual Reality”, ensuring congruence with prevalent terminology in relevant research. To capture recent advancements in VR technology, we limited our search to publications between January 2015 and September 2024. In addition to the aforementioned criteria, each database has specific additional constraints: for Google Scholar, the search keywords must appear in the title; for PubMed, the article types must be Original Article, Clinical Trial, or Randomized Controlled Trial; and for WOS, only articles categorized as ‘Article’ are eligible.

### 2.2 Study selection

To be included in this review, studies needed to meet specific criteria: they must (1) capture quantitative viewpoints of a population; (2) focus on the use and evaluation of immersive VR technology (in abstract); and (3) be related to anxiety, depression, ADHD, or other psychological disorders (in abstract). Studies specifically chosen to provide qualitative insights for clinicians were selected to gather practical and professional perspectives on the application of VR in clinical settings. Only articles that have been accepted and published were chosen to ensure the reliability and credibility of our research findings. For practicality, we limited our search to English language articles and acknowledge potential limitations in scope. While focusing on English articles may not cover all available evidence, this limitation is unlikely to have a significant impact on our comprehensive findings.

**Figure 1:**
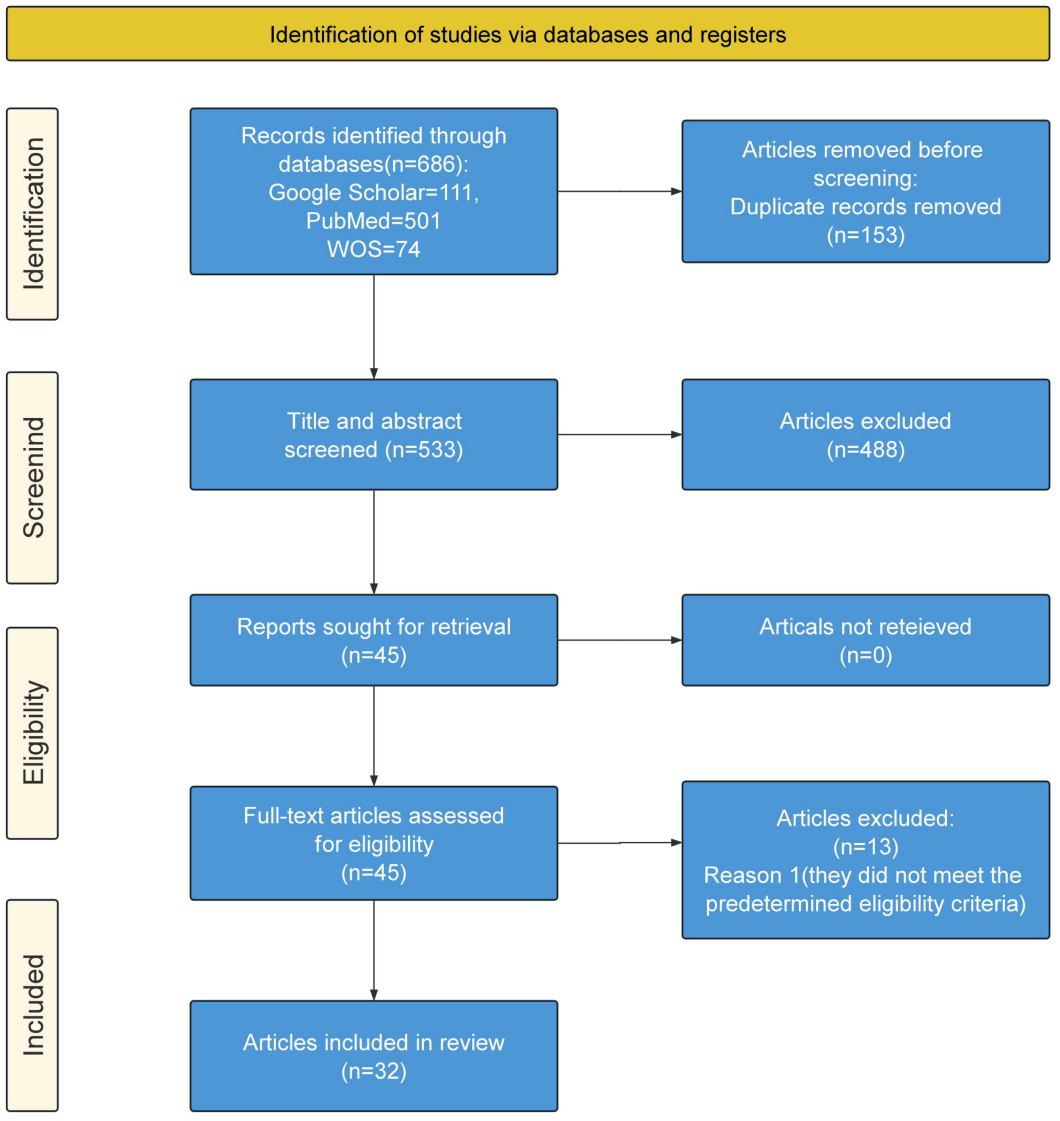
PRISMA 2021 flow diagram.

In the final stage, the search yielded 686 studies. We used the literature management software EndNote to meticulously remove duplicate articles, ensuring each article was uniquely considered. After removing 153 duplicate records, the remaining 533 articles were independently screened based on their titles and abstracts according to predefined inclusion criteria. We conducted a detailed reading of the remaining articles, carefully examining their research content, methods, results, and contributions to the application of VR technology in the field of mental health. Another 488 were excluded, and to maintain fairness and precision in our selection, our team engaged in rigorous, multi-round discussions, sharing personal views, evaluating opinions, and debating controversial articles in depth. This approach led to the final selection of 45 articles for detailed full-text review. During the full-text phase, any differences among authors were extensively discussed until a consensus was reached. As a result, an additional 13 articles were excluded due to non-compliance with established standards, leaving 32 articles considered relevant and suitable for inclusion in this review. The current scoping review follows the Preferred Reporting Items for Systematic reviews and Meta Analysis extension for Scoping Reviews (PRISMA-ScR) guidelines (11). The review selection process is shown in **Figure 1**, using the PRISMA diagram. The research strategy, inclusive of these steps, and its outcomes are presented in **Table 1**. The characteristics of the included studies are charted in **Table 2**.

**Table 1:** Search strategy and results.

Search keywords	Results by year	Number of articles
**VR AND Anxiety**	2015.01-2024.8	WOS (3)、PubMed (350)、Google Scholar (60)
**VR AND Depression**	2015.01-2024.12	WOS (0)、PubMed (105)、Google Scholar (15)
**VR AND ADHD**	2015.01-2024.12	WOS (30)、PubMed (8)、Google Scholar (7)
**VR AND Delusional Disorders**	2015.01-2024.12	WOS (2)、PubMed (3)、Google Scholar (1)
**VR AND ASD**	2015.01-2024.12	WOS (38)、PubMed (10)、Google Scholar (17)
**VR AND CBT**	2015.01-2024.12	WOS (1)、PubMed (25)、Google Scholar (11)

**Table 2:** Characteristics of included studies - data extraction table.

Author, Date, Country	Design, setting and target group	Number of participants	Data collection method	Summary of study results
1. Anderson et al., 2016 (12), USA	Quantitative. Patients with a clinical diagnosis of social anxiety disorder	N = 28	Experiments & questionnaires	VR exposure therapy and exposure group therapy for social anxiety disorder produce long-lasting benefits, consistent with research on a variety of forms of short-term cognitive behavioral therapy for social anxiety disorder.
2. Stamou et al., 2021 (13), Spain	Mixed Methods. Women referred by a GP or other health/mental health provider in Dunedin, New Zealand for postnatal depression	N = 15	Questionnaires	Results show that the combination of CBT with VR is an effective treatment for PND. In addition, VR can enhance awareness, decision-making, and self-appreciation within the individual and can also have real-life applications. This study also shows that the combination of VR and CBT is feasible, while the use of such a technology is well accepted.
3. Guitard et al., 2019 (61), Canada	Mixed Methods. Generalized Anxiety Disorder (GAD) patients were recruited through the Université du Québec en Outaouais (UQO) via email and posters.	N = 28	Questionnaires	The results suggest that the standardized virtual scenario induced significant anxiety. No difference was found when comparing exposure to the standardized scenario in VR and exposure to the personalized scenario in imagination. These findings were specific to anxiety and not to the broader measure of negative affect. Individual differences in susceptibility to feel present in VR was a significant predictor of increase in anxiety and negative affect.
4. Geraets et al., 2019 (14), USA	Mixed Methods. Patients with a diagnosis of generalized SAD	N = 15	Questionnaires	This uncontrolled pilot study demonstrates the feasibility and treatment potential of VR-CBT in a difficult-to-treat group of patients with generalized SAD. Results suggest that VR-CBT may be effective in reducing anxiety as well as depression, and can increase quality of life.
5. Bouchard et al., 2017 (15), Netherlands	Quantitative. Participants were recruited through referrals from practitioners at the investigators’ site and advertisements in local newspapers and university networks.	N = 59	Experiments & questionnaires	Using VR can be advantageous over standard CBT as a potential solution for treatment avoidance and as an efficient, cost-effective and practical medium of exposure.
6. Jeong et al., 2021 (16), Korea	Mixed Methods. Among patients with SAD who visited the department of psychiatry in Gangnam Severance Hospital, 115 patients who participated in VR-based CBT were the targets of the retrospective analysis.	N = 115	Experiments & questionnaires	The results suggest that short-term VR-based individual CBT of nine to 10 sessions may be effective. When the therapeutic effect is insufficient during this period, the additional benefit may be minimal if the session is simply extended. The improvement in the early termination group suggests that even shorter sessions of five or six can also be effective.
7. Montesano et al., 2021 (17), Spain	Quantitative. Patients will be recruited at each health center and from an open call through social media. All participants will meet diagnostic criteria for a mild or moderate depressive episode	N = 225	Experiments & questionnaires	This study provides evidence for PCT-VR, expanding the range of evidence-based technological psychotherapy interventions for young people and helping to prevent the progression of disease worsening.
8. Freeman et al., 2016 (18), UK	Quantitative. Thirty patients with persecutory delusions were recruited from adult mental health services in Oxford Health NHS Foundation Trust.	N = 30	Experiments	In comparison with exposure, **virtual reality** cognitive therapy led to large reductions in delusional conviction (reduction 22.0%, P = 0.024, Cohen’s d = 1.3) and real-world distress (reduction 19.6%, P = 0.020, Cohen’s d = 0.8). Cognitive therapy using VR could prove highly effective in treating delusions.
9. Liao et al., 2018 (19), China	Quantitative. Ten participants were recruited.	N = 10	Experiments	The study successfully designed interactive scenarios for depression assessment in a VR environment, following a specific route of design element analysis. Preliminary evaluations by normal users confirmed a good user experience.
10. Jeppesen et al., 2022 (20), Denmark	Quantitative. Participants were recruited from the outpatient routine care settings of patients suffering from schizophrenia spectrum disorders in the Capital Region of Denmark and the North Denmark Region.	N = 256	Experiments	The trial will elucidate whether VR-CBT can enhance therapy efficacy for paranoid ideations. Additionally, Trial findings will provide evidence on the effectiveness and cost-effectiveness of VR-CBT for paranoid ideations that can guide the possible dissemination and implementation into clinical practice.
11. Pot-Kolder et al., 2018 (21), Netherlands	Quantitative. Participants were recruited from the outpatient clinics of seven mental health centers in the Netherlands.	N = 116	Experiments	The results suggest that the addition of VR-CBT to standard treatment can reduce paranoid ideation and momentary anxiety in patients with a psychotic disorder.
12. Geraets et al., 2020 (22), Netherlands	Quantitative. Participants were recruited from seven mental health centers.	N = 91	Experiments	VR-CBT reduced paranoid symptoms and lowered levels of negative affect in daily life, but did not affect the extent to which mental states influenced each other. Findings do suggest that as a result of treatment mental states regain flexibility.
13. Chu L et al., 2023 (23), China	Quantitative. Preschool children diagnosed with ASD	N = 78	Experiments	They found potentially positive effects of nonwearable digital therapy plus LSP on core symptoms associated with ASD, leading to a modest improvement in the function of sensory, motor, and response inhibition, while reducing impulsivity and hyperactivity in preschoolers with both ASD and ADHD. VR-CBT was found to be an effective and feasible adjunctive digital tool.
14. Donker et al., 2019 (23), Netherlands	Quantitative. The general Dutch population who experience symptoms of acrophobia and use Android smartphones.	N = 193	Experiments & questionnaires	A low-cost fully self-guided app-based **virtual reality** cognitive behavioral therapy with rudimentary VR goggles can produce large acrophobia symptom reductions.
15. Clemmensen et al., 2020 (24), Denmark	Quantitative. Patients are recruited from the mental health services in the Region of Southern Denmark and from the Internet clinic at the Centre for Telepsychiatry (CTP).	N = 90	Experiments	Positive findings will support the use of CBT with VR-based exposure as an alternative, or supplement, to *in vivo* exposure.
16. Hildebrand et al., 2022 (25), Germany	Quantitative. Patients with SAD will be recruited from the general population and routine care at the Outpatient Center for Psychotherapy of the University of Siegen, Germany.	N = 40	Experiments	Self-guided digital therapeutic applications including ultra-short-time therapy combined with VR could help reduce the waiting time for patients and relieve the health system.
17. Kampmann et al., 2016 (26), Netherlands	Quantitative. Participants were recruited via online and newspaper advertisements, the website of the ambulatory of the University of Amsterdam, and the project’s website.	N = 60	Experiments	VR exposure therapy containing extensive verbal interaction without any cognitive components can effectively reduce complaints of generalized social anxiety disorder. Future technological and psychological improvements of virtual social interactions might further enhance the efficacy of VRET for social anxiety disorder.
18. Arnfred et al., 2022 (27), Denmark	Quantitative. Patients with a diagnosis of social anxiety disorder and/or agoraphobia from the regional mental health centers of Copenhagen and North Zealand and from the northern part of Denmark.	N = 302	Experiments	The study was embedded in an outpatient hospital setting, so the intervention design was flexible. This increases ecological validity but also increases the risk of systematic bias in treatment management.
19. Kiper et al., 2022 (28), Italy	Quantitative. Inclusion criteria were 55-75 years of age, a history of ischemic stroke and a 30-item Geriatric Depression Scale (GDS-30) score of ≥10.	N = 60	Experiments	The use of VR therapy combined with neurological rehabilitation had a positive effect on improving mood and reducing depressive symptoms in post-stroke patients.
20. Veling et al., 2021 (29), Netherlands.	Quantitative. Patients receiving outpatient care at the Department of Psychiatry at the University Medical Center Groningen (UMCG), the Lentis Center for Integrative Psychiatry, or one of the participating local general practices were eligible for the study.	N = 50	Experiments	Both the VR relaxation exercise and the standard relaxation exercise led to immediate statistically significant improvements in all negative and positive affective states. Compared to standard relaxation, the VR relaxation exercise led to significantly greater reductions in total negative affective states.
21. Beidel DC et al., 2021 (30), USA	Quantitative. Recruitment occurred through digital media, flyers and attendance at child-themed events in the Central Florida area (e.g., science museum events). In order to participate in the study, the child had to be between 7 and 12 years old and have a primary diagnosis of SAD.	N = 105	Experiments	VR therapy is a viable, effective and easily disseminated element of a comprehensive treatment program for children with social anxiety disorder.
22. Kim et al., 2022 (31), Korea	Quantitative. Social Anxiety Disorder Patients	N = 52	Experiments	The increased ability induced by VR self-training to unapologetically attend to social stimuli and even positively regulate emotional cues is based on functional changes in the visual cortex and thalamus. Based on these short-term neuronal changes, VRS could be a preferred intervention option for SAD patients who are socially avoidant or unwilling to undergo formal treatment.
23. Tabrizi et al., 2020 (32), Iran	Quantitative. The population consists of 2018-2019 year old elementary school students with ADHD in Isfahan city during the 7-12 school year.	N =48	Experiments	VR therapies and medications appear to be effective in improving memory in elementary school children, and the effects of these treatments are still in the follow-up phase, but treatments in VR are more effective than medications in both the posttest and follow-up phases.
24. Cho Y et al., 2024 (33), Korea	Quantitative. Adults were co-recruited from the community. Participants with a score of ≥10 on the Patient Health Questionnaire-9 (PHQ-9) or ≥9 on the Panic Disorder Severity Scale (PDSS) were included in the Depression or Anxiety Symptoms group (DAS group), while others were categorized as the Healthy Control group (HC group).	N =120	Experiments	VR-based BF was effective in reducing depressive and anxiety symptoms, even for subthreshold depression and anxiety symptoms in the healthy control group.
25. Rodrigo-Yanguas et al., 2021 (34), Spain	Quantitative. Patients aged 12-22 years with pharmacologically stable ADHD were enrolled.	N = 105	Experiments	This is the first study testing an augmentation strategy using either a serious videogame or chess in clinically drug-treated patients with ADHD. Using VR serious videogames present with several advantages over traditional
26. Mühlberger et al., 2020 (35), Germany	Quantitative. Performance of 94 children with ADHD (n = 26 treated with methylphenidate, n = 68 unmedicated) and n = 34 healthy children on a continuous performance test within a VR classroom (CPT-VRC).	N = 94	Experiments	VR is a promising technology for assessing ADHD symptoms in an ecologically valid environment.
27. McKay et al., 2022 (36), Australia	Quantitative. Adolescents with elevated ADHD symptoms were recruited from secondary schools and ADHD organizations located in Victoria, Australia.	N = 100	Experiments	The intervention used in this study was a novel VR program designed to train inhibitory control in adolescents with elevated symptoms of ADHD.
28. Eom et al., 2019 (37), Yonsei	Quantitative. 18 typically developing children (TDC) and 20 ADHD children and adolescents	N = 38	Experiments	Performance in the VR-CPT program was associated with behavioral measures of ADHD symptoms. Adding social aspects to a VR environment commonly encountered by children and adolescents has the potential to make a difference in the attention performance of youths with ADHD.
29. Celestino Rodríguez et al., 2018 (38), Spain	Quantitative. A total of 241 (71.30%) boys and 97 (28.70%) girls aged between 6 and 16 (M = 10.84, SD = 3.01) participated in the study, with an average IQ of 104.11 (SD = 11.85).	N = 338	Experiments	Results indicated that VR-CPT predicts ADHD presentations better than TOVA. It also differentiates better between ADHD and non-ADHD students.
30. Bioulac et al., 2020 (39), France	Quantitative. Children with ADHD	N = 51	Experiments	This study demonstrates for the first time that a cognitive remediation program delivered in a virtual classroom reduces distractibility in children with ADHD and could replace methylphenidate treatment in specific cases.
31. Hong et al., 2022 (40), Korea	Quantitative. 21 ADHD, 19 controls	N = 40	Experiments	VR is possibly a useful tool for investigating the effect of distractors on individuals with ADHD, and children with ADHD are more vulnerable to a low-level stimulation situation than normal children in VR.
32. Wang et al., 2024 (44), China	Quantitative. Undergraduate students from Tianjin Medical University served as participants in the study.	N = 68	Experiments	The incorporation of distractors in VR-CPT modulates EEG signals across different frequency bands, with visual distractors attenuating theta band activity, auditory distractors enhancing alpha band activity, and both types of distractors reducing beta oscillations following target stimuli. This insight holds significant promise for the rehabilitation of children and adolescents with attention deficits.

## 3 Results

### 3.1 Applications of VR in anxiety and depression

In recent years, the application of VR technology in the field of mental health has garnered increasing attention, particularly in the treatment of anxiety and depression (24, 25, 27–29, 31). By simulating various real-world scenarios, VR technology provides individuals with a safe and controllable environment, enabling them to confront and process anxiety triggers without actual risk. The combination of VR technology with CBT has demonstrated significant effectiveness.

A study by Anderson et al. (12) explored the application of VR in treating public speaking anxiety. Participants were asked to deliver speeches in virtual conference rooms, classrooms, and auditoriums with an increasing number of audience members. This simulated environment allowed individuals to gradually adapt to and overcome their anxiety in a safe setting. Stamou et al. (13) investigated the use of VR in treating postpartum depression. They created a virtual home environment where participants organized virtual rooms while being exposed to virtual stress. This intervention aimed to help participants learn how to cope with stress in daily life and potentially alleviate symptoms of postpartum depression.

Geraets et al. (14) evaluated the feasibility and potential impact of VR-CBT for patients with severe generalized Social Anxiety Disorder (SAD). This study demonstrated the feasibility and therapeutic potential of VR-CBT in treatment-resistant patients with generalized SAD. The results suggest that VR-CBT may effectively reduce anxiety and depression, and improve quality of life. Cho Y et al. (33) investigated the effects of virtual reality-based biofeedback (VR-BF) on symptoms of depression and anxiety, finding that VR-BF can effectively alleviate symptoms of depression and anxiety. Additionally, research by Bouchard et al. (15) showed that VR-CBT treatment significantly reduced anxiety levels and improved social functioning among patients with social anxiety. Jeong et al. (16) found that for patients with social anxiety disorder, individualized CBT based on VR showed improvement effects within nine to ten short-term sessions, and the additional benefits from extended sessions may be limited, indicating that short-term treatment may be an effective treatment approach.

Beidel et al. (30) demonstrated that VR therapy is a viable, effective and easily disseminated element of a comprehensive treatment program for social anxiety disorder in children. Another study by Kampmann et al. (26) also confirmed the effectiveness of VR exposure therapy in reducing symptoms of social anxiety disorder. The study by Cho Y et al. (33), on the other hand, showed that VR-based behavioral activation (BF) was effective in reducing depression and anxiety symptoms, even in healthy controls with subthreshold depression and anxiety symptoms. Together, these studies highlight the potential of VR technology in mental health interventions, particularly in providing more accessible and personalized treatment options.

It is noteworthy that VR-CBT treatment is not only beneficial for patients but also more practical and efficient for therapists. VR technology enables therapists to easily simulate various complex scenarios, providing personalized treatment plans for patients. Furthermore, VR technology can record patients’ behavioral responses and physiological indicators, providing therapists with more comprehensive evaluation bases.

### 3.2 Applications of VR in ADHD

ADHD is a common neurodevelopmental condition affecting millions of children and adults worldwide. In the United States, data from the Centers for Disease Control and Prevention (CDC) indicates that approximately 9.8% of children aged 3-17 have received an ADHD diagnosis. Characterized by a triad of core challenges – inattention, hyperactivity, and impulsivity – ADHD can significantly impact daily life, causing difficulties with focus, organization, emotional regulation, and social interaction (41). Effective treatment options are crucial for managing ADHD symptoms and improving overall well-being. Traditionally, ADHD treatment has relied on a combination of medication and behavioral therapy, such as CBT (42). However, the field of mental health is constantly evolving, and new technologies are emerging with the potential to enhance existing treatment approaches.

Eom et al. investigated attention performance in children within a virtual classroom and demonstrated that Virtual Reality-Continuous Performance Test (VR-CPT) is an effective method for assessing attention capabilities. They found that the presence of virtual characters can influence the attention performance of children with ADHD (37). Further research is needed to explore how the number of students in virtual classrooms affects attention abilities. In 2018, Celestino Rodríguez et al. compared traditional and virtual classroom methods for continuous testing in identifying ADHD. They introduced visual, auditory, and audiovisual distractions to enhance ecological validity and examined variables such as omission errors, commission errors, and reaction time. The results indicated that VR is more predictive of ADHD performance than traditional continuous tests and better distinguishes between students with and without hyperactivity disorders (38). Virtual classrooms offer a more realistic and ecologically valid assessment environment compared to traditional neuropsychological measures. Bioulac et al. demonstrated that this approach effectively improves cognitive functions, including ADHD symptoms and targeted cognitive skills (39). However, cognitive remediation as a non-pharmacological treatment for ADHD may have adverse effects, poor compliance, or lead to negative attitudes toward medication (43). Hong et al. examined the effects of distracting factors in a virtual classroom on the attention and hyperactivity of children and adolescents with ADHD. They compared behavioral data and head movements in VR-CPT across two developmental stages, finding that children with ADHD performed comparably to controls under distracting conditions but worse without distractions. Additionally, they exhibited more head movements under distracting conditions (40). VR is a useful tool for studying the impact of distractions on individuals with ADHD, and understanding different responses to various distractions is crucial for targeted interventions. Wang et al. studied the effects of visual and auditory distractions on electroencephalography (EEG) in a virtual classroom and found that these distractions modulated brain signals in different frequency bands, with visual distractions reducing theta band activity and auditory distractions enhancing alpha band activity (44).

A study by Mühlberger et al. (35) showed the potential of VR technology for assessing ADHD symptoms in an ecologically valid environment, which could help to more accurately understand patient performance and needs. Rodrigo-Yanguas et al. (34) were the first to develop a study protocol to test the use of serious video games or chess as an augmentation strategy for clinically medicated ADHD patients, such that VR technology could have innovative application prospects in ADHD treatment. Tabrizi et al. (32) found that VR therapy and medication are effective in improving memory in elementary school students, with VR treatment showing superior effects than medication in both post-test and follow-up phases. The study of McKay et al. (36) indicates that VR technology has broad application prospects in the assessment and treatment of ADHD, particularly in providing more personalized and effective intervention measures. VR represents a promising trend with substantial potential for predicting, assessing, and intervening in ADHD.

### 3.3 Applications of VR in other mental illnesses

VR-CBT has demonstrated diagnostic and therapeutic efficacy comparable to, or even superior to, traditional CBT for mental health conditions such as depression, delusions, and phobias (10, 18). In diagnosing and treating depression, VR-CBT offers multi-sensory stimulation by simulating realistic situations, providing better immersion and interactivity compared to traditional CBT (19). For schizophrenia spectrum disorders, VR-CBT enhances exposure and behavioral experimentation in a safe virtual environment, leading to significant treatment improvements. Jeppesen et al. (20) conducted a randomized clinical trial comparing VR-CBT to standard CBT in 256 patients. The results indicated that VR-CBT produced a more effective behavioral component, which is crucial for treating delusional disorders. Pot-Kolder et al. (21) performed a single-blind, randomized controlled trial comparing VR-CBT with a control therapy for delusional disorders and social avoidance in psychiatric patients. The VR-CBT group experienced significant reductions in momentary paranoia and anxiety compared to the control group, with these improvements maintained at follow-up. Similarly, Geraets et al. (22) found VR-CBT more effective in improving paranoia and negative affect compared to conventional treatment, though it did not significantly impact positive affect. Chu et al. found that VR-CBT had a potentially positive impact on core symptoms associated with autism spectrum disorder (ASD), leading to modest improvements in sensory, motor, and response inhibition functioning, as well as reductions in impulsivity and hyperactivity in preschoolers with ASD and ADHD (23). Overall, VR-CBT has proven to be an effective and viable augmentative digital tool.

Recent evidence suggests that CBT has a consistently small to moderate efficacy in reducing paranoia (9). To enhance its effectiveness, targeted treatments with efficacy approaching the moderate range is recommended (45). Achieving an appropriate balance between behavioral and cognitive therapies is crucial; however, behavioral treatments face challenges due to difficulties in organizing and controlling exposures that stimulate delusional ideation in real-life settings. The efficacy of CBT for treating psychotic symptoms can be further improved through the integration of VR technology. VR exposures, being symptom-specific, offer better control over the behaviors of individuals with delusional disorders, making VR-CBT a promising approach for transitioning from research trials to real clinical settings. Recent meta-analyses indicate that VR exposure therapy is comparable in effectiveness to traditional exposure therapy (46). Donker et al. (46) developed an app-based VR-CBT specifically for treating agoraphobia and conducted a randomized clinical trial that demonstrated this low-cost, app-based VR-CBT significantly reduced symptoms of agoraphobia.

Multiple studies have demonstrated the effectiveness of VR-CBT in treating mental illness, particularly in enhancing the crucial behavioral component for effective treatment. Notably, VR has the ability to capture momentary experiences, including thoughts, emotions, and actions, thereby unlocking vast therapeutic possibilities that have been underutilized.

### 3.4 The rise of AI in VR-CBT

In recent years, the integration of AI technology into VR-CBT has emerged as a promising trend in mental health treatment (47–50). This fusion of AI and VR-CBT offers unprecedented opportunities to enhance mental health outcomes through more precise, adaptive, and accessible interventions. Li et al. (48) employed deep learning algorithms to assess depression and anxiety levels, providing an automated assessment method for mental health evaluations via human-computer interaction and VR. Egan et al. (49) found that CBT effectively reduces perfectionist tendencies and associated psychopathological symptoms. Their study on young people’s perceptions of an AI-assisted CBT intervention revealed positive attitudes, with the AI-assisted CBT being perceived as accessible and cost-effective. Horesh et al. (50) demonstrated that integrating VR with AI can alleviate hot flash symptoms and improve mental health in women with breast and ovarian cancer, highlighting the potential for combining AI with VR-CBT to provide personalized psychotherapy. With AI integration, VR-CBT can dynamically adapt treatment protocols based on real-time patient data, enhancing both treatment efficacy and personalization. AI algorithms can analyze user interactions within virtual environments, monitor physiological responses, and deliver targeted interventions tailored to individual needs (51, 52).

However, despite its therapeutic potential, VR-CBT faces technical challenges. AI integration is still in its early stages, requiring extensive data collection and refinement of algorithms. Further research is essential to validate the long-term efficacy of VR-CBT, and the lack of consistent technical standards may impede its broader adoption in clinical practice.

### 3.5 How AI can enhance VR-CBT

AI has the potential to significantly enhance VR-CBT by analyzing user data within the virtual environment, including performance and physiological responses, to dynamically tailor the treatment process (53). For instance, AI can adjust task difficulty based on user performance or modify elements of the virtual environment according to anxiety levels. The integration of AI technology offers critical opportunities for personalized, adaptive, and data-driven interventions. AI algorithms can process extensive patient data collected during VR-CBT sessions, such as physiological responses, behavioral patterns, and user interactions within virtual environments. By employing machine learning and natural language processing techniques, AI can customize treatment protocols to meet the specific needs and preferences of individual patients. Additionally, AI-powered virtual therapists can provide real-time feedback, guidance, and support during VR-CBT sessions, thereby enhancing therapeutic engagement and efficacy. Furthermore, AI-driven data analytics can reveal insights into treatment outcomes, supporting the continuous improvement and optimization of VR-CBT interventions.

VR-CBT represents a promising avenue for innovative, effective, and practical treatments in mental health. To further validate its effectiveness and cost-efficiency, future research should address technical challenges and conduct large-scale, methodologically rigorous controlled studies. These efforts are crucial for facilitating the widespread adoption of VR-CBT in clinical practice.

### 3.6 Novel AI-powered CBT-CHAT framework: revolutionizing the enhancement of cognitive behavioral therapy

The proposed CBT-CHAT treatment framework enhances CBT by integrating advanced AI technologies. Through CBT-CHAT, patients undergo eight structured CBT treatment sessions. This AI-driven system autonomously generates dialogue content in line with established CBT standards, ensuring consistency and efficacy while significantly reducing clinicians’ time. As the system interacts with more patients, it becomes increasingly effective in managing various mental health conditions.

**Figure 2:**
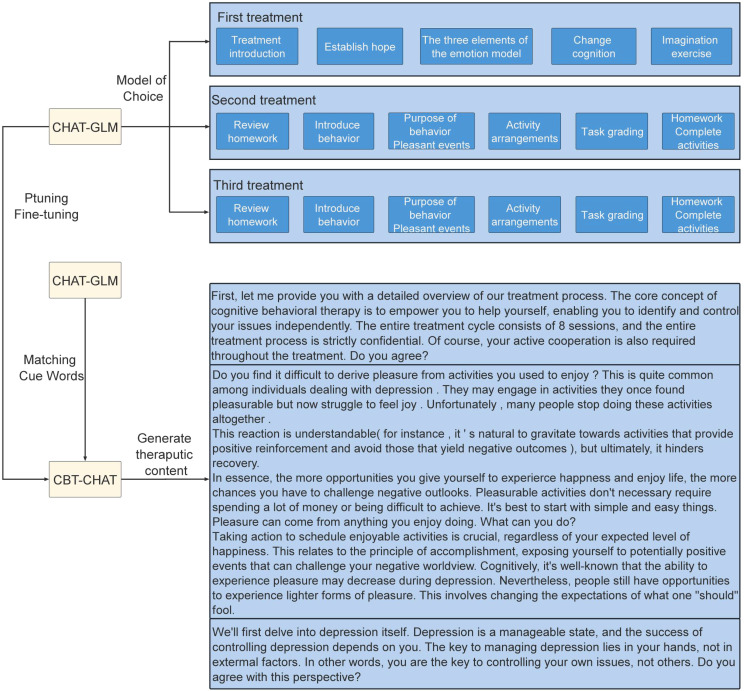
CBT-CHAT treatment framework.

**Figure 3:**
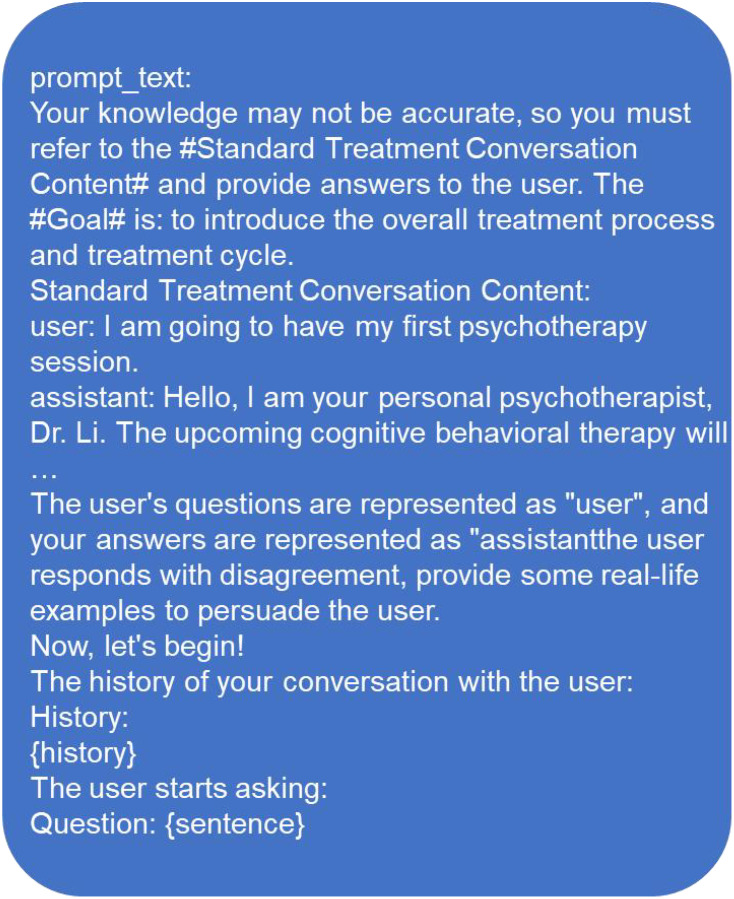
First treatment prompt.

The CBT-CHAT framework, depicted in **Figure 2**, innovatively incorporates LLMs into CBT. This cutting-edge approach utilizes the CHAT-General Language Model (CHAT-GLM) and CBT-CHAT models, with the latter fine-tuned using the pruning technique on a bespoke dataset to align with CBT principles. Pruning refines the model parameters with minimal CBT-specific data, allowing for tailored responses. The CHAT-GLM model initially matches patient responses with appropriate treatment modules and prompt words, as illustrated in **Figure 3** for the first session. Subsequently, the CBT-CHAT model generates relevant CBT content based on these prompts and the patient’s inputs, aiming to personalize and optimize the therapeutic process.

The treatment process spans eight sessions, with the first three outlined as follows:

#### 3.6.1 First treatment session

Focuses on introducing the treatment and fostering hope in the patient. The therapist provides an overview of the core concepts and goals, ensuring the patient understands the treatment process and anticipated outcomes. The patient learns the three elements of the emotion model, aiding in understanding the formation and impact of emotions. Cognitive restructuring techniques help the patient to identify and alter negative thoughts. Imagination exercises are used to envision positive outcomes, enhancing confidence and motivation.

#### 3.6.2 Second treatment session

Emphasizes reviewing homework, introducing new behaviors, and discussing their purposes. The therapist reviews the patient’s homework, summarizing experiences and lessons learned. New behavior patterns are introduced, and their positive impacts are explained. The session focuses on arranging pleasant activities to evoke positive emotions. Task grading helps the patient to assess their performances, and new homework is assigned to encourage specific activities.

#### 3.6.3 Third treatment session

Continues with a review of homework, further introduces new behaviors, and arranges activities. The therapist reviews the patient’s homework, assisting in analysis and improvement. New behavior patterns and activity arrangements are introduced to ensure continuous engagement with positive events. Task grading and additional homework reinforce the therapeutic process, ensuring that the patient sustains these positive changes.

By leveraging this structured approach, the AI-driven CBT-CHAT framework enhances treatment outcomes and offers a scalable, adaptable solution for mental health care. Future research should validate this approach through rigorous clinical trials and explore its long-term benefits. Addressing these challenges will pave the way for broader adoption of this technology in clinical settings, ultimately improving access to high-quality mental health care.

## 4 Discussion

The primary objective of this review is to evaluate the effectiveness of VR technology in diagnosing and treating mental disorders, such as anxiety, depression and ADHD. Additionally, we introduce the CBT-CHAT model framework, which is designed to enhance CBT.

To assess the effectiveness of VR technology in diagnosing and treating anxiety disorders, depression, and ADHD, we compiled relevant literature from the past decade. The results indicate that, compared to control groups, VR-based interventions have significantly improved outcomes for anxiety, depression, and ADHD (39, 54). Similarly, two meta-analyses report that immersive VR interventions have significantly enhanced sustained attention and vigilance in children with ADHD (55–58). However, research on the effectiveness of VR in diagnosing and treating depression and anxiety is relatively scarce, particularly in large-scale validation. Further studies are needed to enhance our understanding of VR’s role in diagnosing and treating anxiety and depression. Additionally, a review highlighted the growing interest in using immersive technologies such as VR and augmented reality, especially VR, for treating depression (59). While VR holds significant potential for enhancing mental health care, its successful integration into clinical practice necessitates addressing existing gaps in knowledge, training, and structural support (60).

Although VR has shown promising results in treating anxiety, depression, and ADHD, it is essential to acknowledge its limitations. A significant limitation is the high cost of VR equipment and software, which may limit its access for many patients, particularly those in low-income groups or underfunded healthcare settings. Additionally, some users may experience technical issues such as motion sickness or discomfort, potentially hindering the effectiveness of VR interventions. Another limitation is the need for specialized training for therapists to effectively administer VR-based treatments. This requirement can pose challenges to widespread adoption and implementation. Finally, although VR offers immersive therapeutic environments, it may not fully replicate real-life scenarios, possibly limiting the transfer of skills or benefits gained during VR sessions to everyday life. Despite these limitations, ongoing advancements in VR technology and research continue to address these challenges, making VR an increasingly viable option for mental health interventions.

Regarding the application of AI combined with VR technology in mental health treatment, we searched for studies exploring the combination of AI and VR to address psychological and physiological health issues. Although related research is limited, existing studies suggest that the combination of AI and VR shows promise in treating psychological and physiological disorders. AI technology has the potential to significantly enhance the effects of VR-CBT by providing personalized, adaptive, and data-driven interventions. AI algorithms can analyze vast amounts of patient data collected during VR-CBT sessions, including physiological responses, behavior patterns, and user interactions within virtual environments. By leveraging machine learning and natural language processing, AI can tailor treatment plans according to individual patient needs and preferences. Thus, the proposed CBT-CHAT framework enhances CBT by integrating advanced AI technologies, becoming more effective as it interacts with more patients. However, the integration of AI with VR-CBT is still in its early stages and requires extensive data collection and algorithm improvement.

This scoping review has several strengths. First, we have exclusively focused on anxiety, depression, and ADHD, providing targeted analyses that offer valuable insights for clinicians and researchers working in these areas. Additionally, we propose that the CBT-CHAT model framework can enhance CBT. Finally, we also analyzed and provided prospects for the application of AI and VR in mental illnesses. However, it is necessary to acknowledge the limitations of our study. First, our literature search was limited to three electronic databases. Although PubMed, Google Scholar, and WOS are comprehensive resources, other databases such as Cochrane Library, EMBASE, etc., might contain relevant studies that were not included in our search scope. Future reviews could benefit by expanding search parameters to cover these additional sources. Second, our review focuses specifically on anxiety, depression, and ADHD. Although these are common mental disorders with considerable clinical relevance, many other mental health conditions may benefit from VR-based interventions. Therefore, future research should consider exploring the application of VR technology in treating a broader range of mental health issues. Third, we only considered English publications. Finally, each study’s virtual environment was different, requiring more studies with larger sample sizes and more consistent outcome measurement indicators for a more systematic review of the same virtual environments and diseases.

## 5 Conclusion

In conclusion, this review demonstrates that immersive VR interventions effectively improve mental health outcomes and should be incorporated into clinical practice. Additionally, integrating AI with immersive VR shows significant potential for enhancing cognitive rehabilitation. This innovative combination can deliver personalized, adaptive, and targeted interventions, further advancing cognitive rehabilitation and improving the quality of life for those in need.

## Data Availability

The original contributions presented in the study are included in the article/supplementary material. Further inquiries can be directed to the corresponding author.
